# Neutrophil-to-Lymphocyte Ratio, Platelet-to-Lymphocyte Ratio and Complete Blood Count Components in the First Trimester Do Not Predict HELLP Syndrome

**DOI:** 10.3390/medicina55060219

**Published:** 2019-05-28

**Authors:** Giovanni Sisti, Andrea Faraci, Jessica Silva, Ruchi Upadhyay

**Affiliations:** Lincoln Medical and Mental Health Center, Department of Obstetrics and Gynecology, Bronx, NY 10451, USA; andrea.faraci@nychhc.org (A.F.); silvaj6@nychhc.org (J.S.); ruchi.upadhyay@nychhc.org (R.U.)

**Keywords:** HELLP syndrome, NLR, PLR, fetal outcome, maternal outcome

## Abstract

*Background and Objectives:* Neutrophil-to-lymphocyte ratio (NLR), platelet-to-lymphocyte ratio (PLR), mean platelet volume (MPV), and other components of the routine complete blood count (CBC) were found to be sensitive biomarkers of preeclampsia and other inflammatory obstetric conditions in previous studies, with conflicting results. We speculated that the same associations existed with hemolysis, elevated liver enzymes, and low platelets (HELLP) syndrome in the first trimester of pregnancy. *Materials and Methods:* We conducted a retrospective case–control study at a tertiary care hospital in NY (USA), in the time frame between January 2016 and December 2018. Our population consisted of pregnant women in the first trimester: We compared patients with HELLP syndrome (cases) with healthy patients (controls) matched by age, body mass index (BMI), parity, and race. Patients with preeclampsia, infection, and fever were excluded. Venous blood samples were obtained as part of the routine work-up during the first prenatal visit in the first trimester, which includes a CBC. The main outcomes were NLR and PLR, and the secondary outcomes were hemoglobin, RDW, platelet count, MPV, neutrophils, and lymphocytes. *Results*: There were 10 patients in each group. There were no differences in NLR and PLR levels and other CBC components between the two groups. *Conclusions*: In our study NLR, PLR, and other CBC components did not predict HELLP syndrome. We speculate that HELLP syndrome has a sudden increase of tissue inflammation in the third trimester that is not manifested during the early phases of placentation. Larger studies are needed to evaluate the true ability of NLR, PLR, and CBC components to predict HELLP syndrome in the first trimester.

## 1. Introduction

Pregnancy-induced hypertensive (PIH) disorders are represented by chronic hypertension, gestational hypertension, preeclampsia, severe preeclampsia, and hemolysis, elevated liver enzymes, and low platelets (HELLP) syndrome [1]. PIH-affected pregnancies carry high mortality and morbidity rates for mothers and fetuses. An early diagnosis is key in order to improve the outcomes.

The neutrophil-to-lymphocyte ratio (NLR) and platelet-to-lymphocyte ratio (PLR) have been proposed as new markers of systemic inflammation [2].

NLR and PLR have been found to be higher in the first trimester in preeclampsia compared to healthy pregnancies [3,4,5,6].

HELLP syndrome has been considered a different entity from preeclampsia by some, even if its proper classification is still a matter of debate [7,8]. HELLP syndrome is part of the hypertensive disorders of pregnancy, but, compared to preeclampsia, the inflammatory reaction is stronger, and the immune system preferentially attacks the liver and the coagulation cascade [7,8,9]. In addition, while hypertension is an invariable factor in preeclampsia, it is not always present in HELLP syndrome and it is not part of the diagnostic criteria [7,8].

While the first trimester inflammatory reaction in preeclampsia has been extensively studied, studies regarding the immune activation specifically for HELLP syndrome are lacking.

We speculated that the same first trimester associations seen in preeclampsia and other inflammatory obstetric conditions in the first trimester existed in HELLP syndrome.

We compared NLR, PLR, mean platelet volume (MPV), hemoglobin, red cell distribution width (RDW), platelet count, neutrophils, and lymphocytes in the first trimester between women with HELLP syndrome and healthy pregnant women.

## 2. Material and Methods

We conducted a retrospective case–control study at a tertiary care hospital in NY (USA), in the time frame between January 2016 and December 2018. The IRB approved the study (IRB # 19-005) on 2nd January 2019. The study compared pregnant women in the first trimester who later developed HELLP syndrome during the third trimester to healthy pregnancies in the first trimester who did not develop HELLP syndrome (controls), matched by age, BMI, parity, and race. 

HELLP syndrome was defined by the Tennessee Classification System diagnostic criteria: Increased LDH > 600 U/L, AST ≥70 U/L, and platelets < 100 × 10(9)/L [10].

We also included patients with partial HELLP syndrome as cases, who require two out of the three classical criteria [11,12].

Patients with preeclampsia, infection, and fever were excluded. Venous blood samples were obtained as part of the routine work-up during the first prenatal visit in the first trimester, which includes a complete blood count (CBC).

Demographics and clinical information were recorded, and included age, body mass index (BMI), gravidity, parity, race, birthweight, gestational age at delivery, and gestational age at CBC collection.

This was the first pilot study in the literature analyzing NLR and PLR in HELLP syndrome, and therefore we were not able to calculate the statistical power and estimate the number of patients needed for the sample size.

The main outcomes were NLR and PLR, and the secondary outcomes were hemoglobin, RDW, platelet count, MPV, neutrophils, and lymphocytes. CBC values were analyzed with the hematology analyzer Beckman Coulter. Statistical analysis was performed with SPSS v. 22.0 (IBM, Chicago, IL, US).

Continuous variables were expressed with the median (25th-75th percentile), and categorical variables were indicated by percentage.

We assessed the normality of data with the Shapiro–Wilk test: For all the variables, the test had a *p*-value below 0.05, indicating that all the data significantly deviated from a normal distribution.

To compare demographics and outcomes between the cases and controls, we used the non-parametric Mann–Whitney test. A *p*-value of less than 0.05 was considered statistically significant.

## 3. Results

There were 10 patients in each group. The median age was 29 (23–31) years old, and the median BMI was 27 (25–29) kg/mt^2^. Race was distributed as follows: 14 Hispanic and six Afro-American. Five out of 20 were multipara. They were matched by age, race, BMI, and parity (Table 1).

Gestational age at delivery (35 vs. 39 weeks, *p* = 0.0139) and birthweight (2575 vs. 3622 gr, *p* = 0.0148) were lower in cases vs. controls.

The median gestational age at blood collection was 5 weeks for cases and 7 weeks for controls. There were no statistically significant differences in NLR (2.2 vs. 2.6) and PLR (131.5 vs. 138.2) levels or other CBC components between the two groups (Table 2).

## 4. Discussion

We did not find any statistically significant differences between NLR, PLR, and other CBC components in the first trimester between HELLP syndrome and healthy patients.

Neutrophil-to-lymphocyte ratio (NLR), platelet-to-lymphocyte ratio (PLR), and other components of the routine complete blood count (CBC) were found to be sensitive early biomarkers of preeclampsia and other inflammatory obstetric conditions in previous studies.

Kirbas et al. [4] found that in the first trimester, NLR was significantly higher in severe preeclampsia compared to healthy pregnancies, and PLR was found to be higher in patients with severe preeclampsia compared to mild preeclampsia.

In a study by Gezer et al. [3], in the first trimester, hemoglobin was lower, and neutrophils, platelets, and PLR were significantly higher in the group with preeclampsia as compared to the control group.

Sachan et al. [6] showed that NLR in the first trimester can predict the occurrence of preeclampsia and severe preeclampsia in the third trimester.

Agrawal et al. [5] found higher lymphocytes, NLR, and PLR in preeclamptic vs. healthy pregnancies. According to our study, NLR and PLR are not good first trimester markers for HELLP syndrome. 

We speculate that, in contrast with preeclampsia, where early placentation is affected and an early state of general inflammation is induced [13], in HELLP syndrome, the immune reaction is unbalanced only in the third trimester or in the postpartum period, and limited to the maternal liver and coagulation system [9].

One strength of our study is to have studied the HELLP syndrome by itself, excluding patients with preeclampsia. In this way, we were able to characterize the unique features of this syndrome and add to its distinction from preeclampsia.

A limitation of this study is the lack of a comparison group of preeclamptic patients, and the low power of the study, given the low number of participants.

## 5. Conclusions

In our study, NLR, PLR, and other CBC components did not predict HELLP syndrome. We speculate that HELLP syndrome has a sudden increase of tissue inflammation in the third trimester that is not manifested during the early phases of placentation. Larger studies are needed to evaluate the true ability of NLR, PLR, and CBC components to predict HELLP syndrome in the first trimester.

## Figures and Tables

**Table 1:** Demographics.

	HELLP (n = 10)	Controls (n = 10)	*p*-Value
Parity > 0	3/10	2/10	NS *
Age (years)	29 (22.2–31)	27 (22.5–30.7)	NS **
BMI (kg/mt^2^)	31.5 (25.2–37.5)	28.3 (23–32)	NS **
Hispanic and Afro-American race	7/10; 3/10	7/10; 3/10	NS *
GA at delivery (weeks)	35 (29–36)	39 (39–39)	**0.0139**
Birthweight (gr)	2575 (1345–2700)	3622 (3370–3691)	**0.00148**

Categorical values are expressed in ratios, and continuous values are expressed in medians (25th–75th percentile). * Chi square test. ** Mann–Whitney test. NS: not significant. HELLP: Hemolysis, elevated liver enzymes, and low platelets. BMI: body mass index. GA: Gestational age. Statistically significant results are expressed in bold.

**Table 2:** Cell blood cell components expressed in medians (25th–75th percentile).

	HELLP (n = 10)	Controls (n = 10)	*p*-Value *
GA at blood collection (weeks)	5 (4–7)	7 (6–10)	NS
Hb (gr/dl)	12.3 (10.1–13.4)	12.8 (11.4–13.5)	NS
Neutrophil (absolute count)	4 (2.8–6)	4.9 (3.6–6)	NS
Lymphocyte (absolute count)	1.9 (1.2–2.8)	1.8 (1.4–2.1)	NS
NLR	2.2 (1.5–2.5)	2.6 (2–3.5)	NS
PLR	131.5 (103–190)	138.2 (105.1–188.2)	NS
Platelet (10^9^/L)	253.5 (230.5–284.7)	270 (207.5–282.7)	NS
RDW (%)	14.1 (13.3–17.4)	13.3 (13–14.1)	NS
MPV (fL)	9 (8.6–10.5)	9.2 (8.3–9.8)	NS

* Mann–Whitney test. NS: not significant. HELLP: hemolysis, elevated liver enzymes, and low platelets. GA: Gestational age; Hb: Hemoglobin; NLR: Neutrophil-to-lymphocyte ratio; PLR: Platelet-to-lymphocyte ratio; RDW: Red cell distribution width; MPV: Mean platelet volume.
